# Avian learning favors colorful, not bright, signals

**DOI:** 10.1371/journal.pone.0194279

**Published:** 2018-03-22

**Authors:** J. P. Lawrence, Brice P. Noonan

**Affiliations:** University of Mississippi, Department of Biology, University, Mississippi United States of America; Universitat Trier, GERMANY

## Abstract

A few colors, such as red and yellow, are commonly found in aposematic (warning) signaling across taxa, independent of evolutionary relationships. These colors have unique traits (i.e., hue, brightness) that aid in their differentiation, and perhaps, their effectiveness in promoting avoidance learning. This repeated use calls into question the influence of selection on specific warning colors adopted by aposematic prey-predator systems. To disentangle the influence of color characteristics on this process, we trained week-old chickens (*Gallus gallus domesticus*) to learn to avoid distasteful food that was associated with one of three color signals (yellow, white, red) that varied in both hue and in brightness in order to assess which of these traits most influenced their ability to learn avoidance. Our results show that while chicks learned to avoid all three colors, avoidance was based on the hue, not brightness of the different signals. We found that yellow was the most effective for avoidance learning, followed by red, and finally white. Our results suggest that while these three colors are commonly used in aposematic signaling, predators’ ability to learn avoidance differs among them. These results may explain why yellow is among the most common signals across aposematic taxa.

## Introduction

Convergent phenotypic evolution, where traits have evolved independently, is widespread in the natural world, and as such, provides an intriguing opportunity to examine the evolutionary pressures driving such patterns. Convergence can be found in a wide variety of taxa from plants [1] and animals [2] to single-celled organisms [3], making the subject of convergent evolution an important question in biology. From the evolution of eyes [4] to flight [5], these “natural experiments” provide perspective into how the natural world functions. Conclusions drawn from these examples can be quite powerful as they represent a broad, repeated pattern from which there are apparent evolutionary mechanisms leading to such convergence. Common explanations for similarity, such as phylogenetic relationships, can be discounted due to the independence of the phenomena, allowing researchers to get to the underlying evolutionary mechanisms that could promote such convergent evolution.

**Fig 1:**
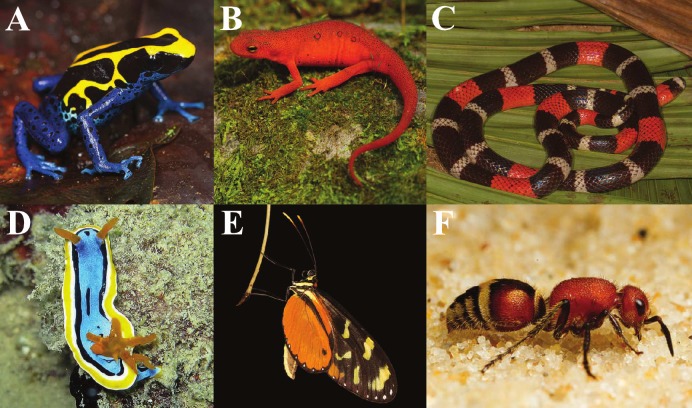
Aposematic signaling has been documented in a wide variety of taxa. These include, but are not limited to, A) Dendrobatid frogs, B) Salamandrid salamanders, C) *Micrurus* coral snakes, D) Nudibranchs, E) *Heliconius* butterflies, and F) Hymenopterans. Among these, similar colors have evolved, which are commonly some variation of yellow, red, and/or white. Printed under a CC BY license, original copyright J.P. Lawrence 2017.

Aposematic (warning) signaling is widespread throughout Animalia (Fig 1), and despite the variety of colors and patterns employed, there are common components to this signaling [6]. Most common are yellow, red, and white coloration, which comprise a dominant component to the vast majority of aposematic signals. Aposematic signaling functions by displaying memorable coloration to would-be predators [7–10]. White, yellow, and red are also among the most contrasting colors against a wide variety of natural backgrounds. These commonalities among diverse aposematic taxa beg the question of why such colors repeatedly appear in independent instances of aposematic signaling. It is important to note that these signals vary not only in color (hue), but also in brightness. It is then possible that the brightness signal components may be important, perhaps more important than hue, in predator perception as this directly affects conspicuousness in a variety of lighting environments.

As research into sensory ecology has progressed, our understanding of the constituent components of signaling has also improved. Aposematic signaling has increasingly been understood to not be comprised solely of conspicuous colors, but rather a complex combination of features that aid in predator recognition, learning, and avoidance [11–13]. Everything from color, pattern, behavior, and lighting environment can impact the learning and recognition of an aposematic signal [14]. Here, we seek to decouple hue from brightness to determine which is more important to the learning of an aposematic signal by predators.

While predators of aposematic species are undoubtedly quite varied and possess a variety of visual systems, birds are commonly used in aposematism studies [15–17]. The primary reasons for this is that they are common predators of aposematic taxa and have tetrachromatic vision, allowing them to perceive and discern color very well [18]. Consequently, we utilized a model avian predator to examine how well naïve individuals can learn to avoid a variety of aposematic signals that vary in both hue and brightness.

## Materials and methods

This research was approved by the University of Mississippi IACUC and conducted under University of Mississippi IACUC 14–026.

### Color analysis

**Fig 2:**
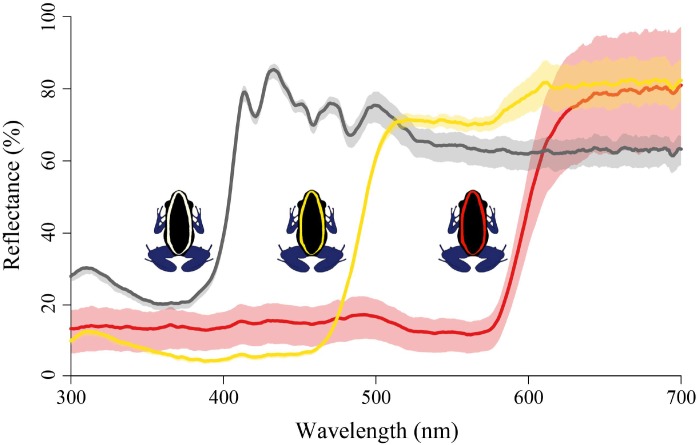
Reflectance curves for each color signal. Color signals were smoothed and averaged using the *pavo* package in R. Lines represent means of all nine measurements for each of the three colors red, yellow, and white (in black) while the shaded region represents a 95% confidence interval for each corresponding signal.

We created three different color signals to test learning. Signals were pictures of drawn frogs with either white, yellow, or red stripes that mimic a pattern found in *Dendrobates tinctorius* (with the exception of red). Bodies were black and legs were blue. White and yellow colors were extracted from photographs of *D*. *tinctorius* from Grand Matoury and Kaw in French Guiana, respectively, while the red color was extracted from photographs of *Oophaga pumilio* from Bastimentos, Panama. These represent colors common in aposematic signaling, but they also represent low (red), medium (yellow), and high (white) brightness (Fig 2). The modeled colors were not meant to exactly match the frogs from which they were extracted, but provide three distinctly different colors varying in both hue and brightness.

We took three color measurements for each color band (see Fig 2) of each printed frog using an Ocean Optics Jaz A3305 spectrometer (S1 Text). We measured three different regions on the color band at the top, middle, and bottom of the band. Measurements were averaged and smoothed in the *pavo* package [19] in R [20] to create reflectance curves for each signal (Fig 2).

We analyzed spectra using the *pavo* package [19] to examine the chromatic (color, hue) and achromatic (luminance, brightness) contrasts between the two colors. The *pavo* package calculates contrasts in Just Noticeable Differences (JNDs) where a value of 1 JND would mean differences could be observed under the modeled vision system when objects are stationary under bright conditions [21]. However, in more natural conditions, signals with 3 JNDs or less are unlikely to be distinguished from one another under a modeled visual system [21].

We used the average avian vision system for visual contrasts and chicken double cone sensitivity to calculate achromatic contrasts under the D65 (standard daylight) illuminant [22]. We conducted all pairwise comparisons between the three measurements for each band. Consequently, each among-band comparison had nine different JND estimates. As JNDs represent a threshold where a modeled visual system can or cannot distinguish signals from one another, we analyzed whether or not means for each signal differ from 1 JND and, a more conservative estimate, 3 JNDs using a nonparametric one-tailed sign test [21].

### Learning experiments

In order to assess how predators learn avoidance, we used one-week-old chickens (*Gallus gallus domesticus*; male cornish rock crosses from the commercial supplier, Ideal Poultry). These were housed in wire brooders (approximately 1m x 1m x 30cm) together while not being tested and fed a commercial corn meal mash. To examine how naïve predators learn color signals, chicks were equally divided (N = 15 per treatment; 45 chicks total) into three different treatments: white signal, yellow signal, and red signal. Trials were done in 30cm x 60cm wooden compartments under full spectrum lighting. Chicks were placed individually into a compartment and allowed to habituate for 2 hours. Chicks were food-deprived during this acclimation period to ensure motivation to feed during the trials.

Once in the compartment and after the 2-hour acclimation period, training consisted of teaching the chicks to eat a dried mealworm (*Tenebrio molitor*) from a petri dish with a printed brown frog on a tan background below the transparent dish. The training phase was completed once the chick had eaten three consecutive times, after which we allowed the birds to rest for a period of 5 minutes.

The avoidance-learning trials consisted of the consecutive presentation of mealworms on a petri dish with a printed frog beneath the dish displaying a warning signal matching the dorsal pattern of *D*. *tinctorius* (described above) with either a white, yellow, or red (these colors hereafter referencing the whole printed frog displayed in Fig 2, not solely the bands) on a tan background. In order to make them unpalatable, mealworms were soaked in a solution of 10% chloroquine for >1 hour. We recorded the hesitation time (i.e., the time until the chick approached and ate the mealworm), and registered any behavioral reaction to the distastefulness of the mealworm after tasted or eaten. Such behaviors most often involved beak wiping and head shaking. Each trial lasted 5min, followed by 5 resting minutes after which a new petri dish with an unpalatable mealworm was offered on the same aposematic background. This procedure was repeated until the chick refused to eat the mealworm over three consecutive trials. The test ended either when chicks “learned” the signal or proceeded through 10 trials, whichever came first. If chicks proceeded through 10 trials without three consecutive refusals, they did not learn the signal. When a chick refused the chloroquine-soaked mealworm on the aposematic pattern, a palatable mealworm was offered on the training (brown frog) background, which the chick did not associate with an unpleasant experience. If the palatable mealworm on a brown frog background was refused (n = 5), these trials were discarded due to the potential for satiation to be confused with learned avoidance. For this reason, trials were run with additional chicks to ensure each treatment had 15 replicates. We registered the number of trials (i.e., mealworms consumed) that it took for the chick to learn to avoid the signal. This research was conducted under University of Mississippi IACUC animal care protocol14-026.

#### Data analysis

We analyzed the data (S1 Table) from the learning experiments using an analysis of variance (ANOVA) in R [20]. We examined the number of attack trials (i.e., how many trials in which chicks tasted the mealworm) and average hesitation time. If ANOVA yielded significant results, we conducted *post-hoc* Tukey’s Honest Significant Difference tests to examine pairwise comparisons among color signals. We examined whether there were differences in chicks’ ability to learn different signals using a generalized linear hypothesis test with Tukey contrasts.

## Results

### Color differences

**Fig 3:**
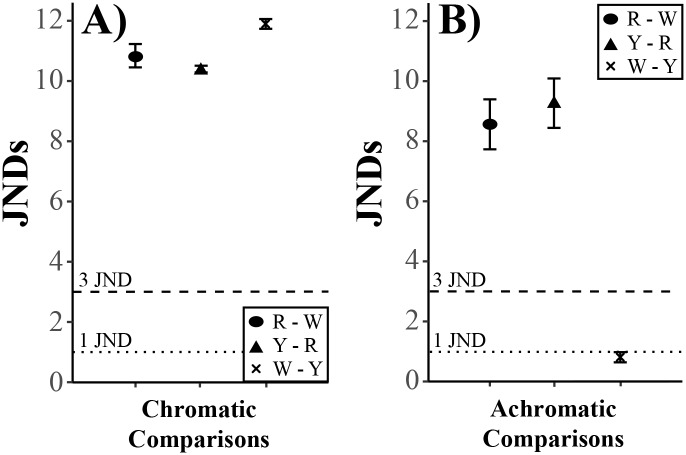
Mean Just Noticeable Differences (JNDs) between different signal comparisons for both A) chromatic and B) achromatic contrasts. Signal comparisons were Red—White (R—W), Yellow—Red (Y—R), and White—Yellow (W—Y). The dotted line represents 1 JND and the dashed line represents 3 JNDs. Error bars represent standard error.

As expected, mean Just Noticeable Differences (JNDs) for each comparison of chromatic contrasts was found to be quite distinct (mean ± SE: red—white: 10.81 ± 0.38, yellow vs. red: 10.37 ± 0.11, and white vs. yellow: 11.86 ± 0.16; Fig 3A). However, achromatic contrasts were distinct between the red—white (mean ± SE: 8.56 ± 0.83) and yellow—red (mean ± SE: 9.27 ± 0.82), but the white—yellow comparison averaged less than 1 JND (mean ± SE: 0.8 ± 0.17; Fig 3B). Nonparametric sign tests for chromatic contrasts revealed that all three comparisons were significantly greater than 1 JND (red—white: p = 0.002; yellow—red: p = 0.002; white—yellow: p = 0.002) and 3 JNDs (red—white: p = 0.002; yellow—red: p = 0.002; white—yellow: p = 0.002). Achromatic contrasts were significantly greater than 1 JND and 3 JNDs for the red—white (1 JND: p = 0.002; 3 JNDs: p = 0.002) and yellow—red (1 JND: p = 0.002; 3 JNDs: p = 0.002) comparisons, but were not significantly greater than 1 JND, and thus 3 JNDs, for the white—yellow comparison (1 JND: p = 0.91; 3 JNDs: p = 1).

### Learning experiments

**Fig 4:**
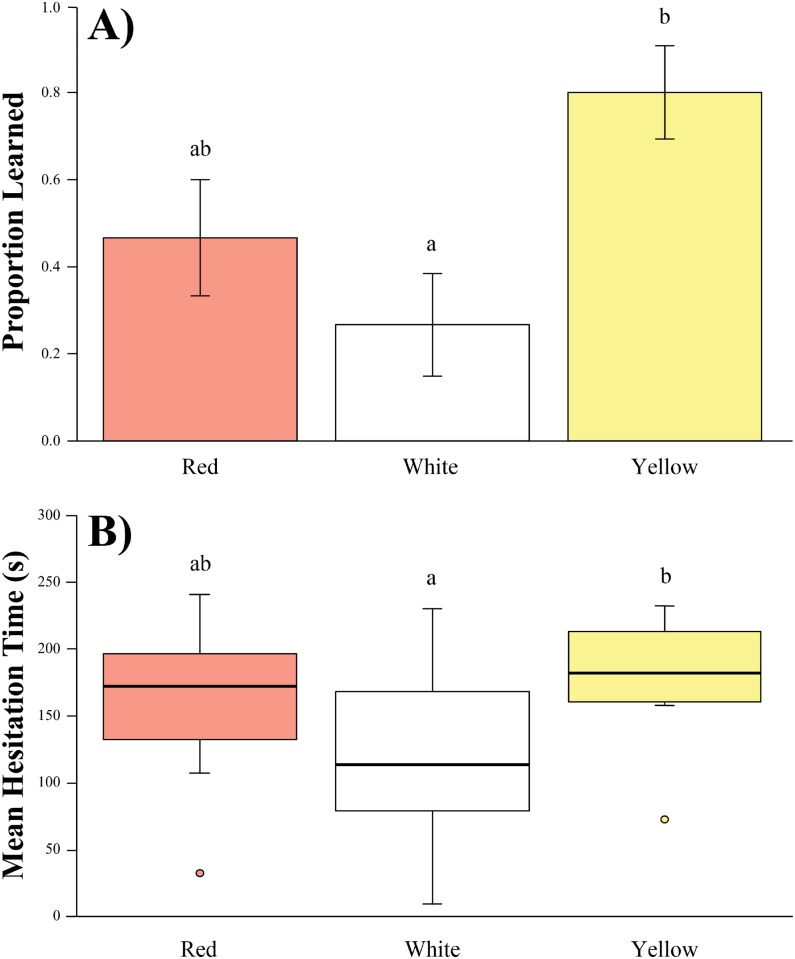
A) Proportion learned and B) mean hesitation time of chicks (*Gallus gallus domesticus*) for each of the three signals. Error bars on the bar graph represent SE. Box plot shows mean, 25^th^ and 75^th^ percentiles of data while vertical lines indicate data range. Circles represent extreme values. Different letters above bars and boxes indicate statistical significance (α = 0.05) from one another.

When examining both the number of trials required to learn avoidance and the average hesitation time, we find significant differences between color treatments in both avoidance trials and average hesitation time (F_2, 42_ = 5.514, p = 0.007 and F_2, 42_ = 5.295, p = 0.009, respectively). Pairwise comparisons yielded significant differences when comparing results from the yellow treatment and white treatment (attack trials: p = 0.005; hesitation time: p = 0.007). While none of the other pairwise comparisons yielded significant differences, the average hesitation time between the red and white treatments did trend towards significant (p = 0.092). When comparing whether there was a difference between signals in how often chicks learned to avoid signals, we found that red was not significantly different from white (p = 0.498) or yellow (p = 0.157). However, when comparing yellow and white, we did find a significant difference (p = 0.016) with chicks more likely to learn to avoid yellow than white (Fig 4).

## Discussion

Many components likely contribute to a predator’s ability to remember and avoid novel warning signals. These can include, but are not limited to, hue, pattern, and brightness. Here, we sought to decouple hue and brightness and determine which is more important at eliciting avoidance behavior. Our results suggest that hue is likely more important than brightness for predator learning, although not all colors are avoided equally. We saw distinct differences between yellow and white signals, with red being intermediate for both learning and hesitation time (Fig 4). Were brightness to be important in determining or limiting predator avoidance, we would have expected white and yellow to be more similar and for both to have evoked greater, or more efficient, learning than red.

Indeed, our results concur with findings previously reported in the literature with regard to hue (color) being the most important factor in determining avian predator learning [23,24]. There may also be an interaction between color and pattern that enhanced avoidance [25], though as we only examined color differences, we are unable to support or refute this. To that end, it is important to note that there are a number of factors that can influence predator learning that are beyond the scope of this study, including lighting environment [14], contrasting backgrounds [26], predator communities [27], etc. Further, our reflectance spectra show some fluorescence in the white signal. While this is likely an artifact of being printed on paper, it is important to note that prior literature suggests that this fluorescence should not affect predator attacks rates between fluorescent and non-fluorescent colors [28]. Despite this, we would encourage some caution in broad application of our results as some color traits are not necessarily broadly applicable to all animals.

While this research was conducted with domestic chickens in a laboratory setting, it is important to give this research context to wild populations and patterns, particularly because prior studies have demonstrated that chicks can be neophobic to novel signals [29]. Interestingly, the pattern observed in our study is mimicked in wild poison frog predator populations where yellow, in general, is better protected than red [26] indicating that patterns observed in the wild are repeatable in laboratory settings. Likewise, several clay model experiments have shown that yellow is especially protective against avian predators [15,26,30] with red providing moderate protection ([31,32]–though importantly, these studies did not examine relative differences of red to other aposematic colors).

This research provides interesting insight into the evolution and proliferation of aposematic signals in the wild. Aposematic signaling is prevalent throughout the animal kingdom, but a number of colors continually evolve independently as signal components (Fig 1). Our results indicate differential learning abilities of avian predators that may explain why we see such signal variation in natural systems. While all three colors did lead to learned avoidance from chickens, the chicks clearly had a more difficult time learning to avoid white, particularly when compared to yellow. As avian predators likely drive aposematic signal evolution in a number of taxa [17,33–35], this could explain why warmer colors (yellow, orange, red) are more commonly used than other, brighter, colors. While birds are capable of seeing into the ultraviolet spectrum, avian visual systems are more sensitive to longer wavelengths [36]. This, coupled with innate biases towards warmer colors [37,38], suggests why warmer colors are prevalent in aposematic signaling.

Of course, our research does raise the question as to why suboptimal signals such as red or white may persist in aposematic species. While we examined the influence of color on aposematic signaling, these colors likely have a wide variety of functions outside of warning signaling. Research suggests that, for example, in the Strawberry Poison Frog (*Oophaga pumilio*) color may be an important component in mate choice [39,40]. Further, aposematic function can be dependent on the distance and behavior of aposematic species [14,41]. At certain distances, counterintuitively, aposematic signals can be disruptive or cryptic. These colors likely have a wide variety of functions that go beyond simple warning signaling, which could explain why suboptimal signals persist and may even be promoted by selection. And finally, suboptimality of aposematic signal should be considered in context of the entire predator community. Avian predators are thought to be important in driving color evolution in poison frogs [22,42,43], however, recent evidence suggests that alkaloid toxin defenses may target insects [44]. As such, while these signals may be suboptimal to avian predators, they may be more effective to other predator taxa.

## Conclusions

Repeated patterns across independent taxa provide an intriguing opportunity to examine underlying evolutionary pressures that may promote such patterns. For example, the repeated banding or longitudinal striping patterns seen in snakes have been shown to have either flicker effects or cause predators difficult in locating vulnerable parts of the body [45]. By dissecting color signals into their constituent parts, we can discern what components are most important for predator decision making. Here, we present evidence that color, not brightness, is important in predator decision making and learning. While our research is limited to a small sample of aposematic colors found in natural systems, it provides important insight into why these colors persist. Further research should focus on why colors, such as yellow, elicit such a strong response in avoidance learning and if this, perhaps, is due to predator physiology (i.e., sensitivity to particular color spectrums) or cognition.

## Supporting information

S1 Text Raw data for color measurements. (ZIP)

S1 Table Raw data for learning trials. (XLSX)
