# Supplementary figures and images for: Avian learning favors colorful, not bright, signals
Source: PLoS One. 2018 Mar 22;13(3):e0194279. doi: 10.1371/journal.pone.0194279 (PMC5864004; doi:10.1371/journal.pone.0194279)

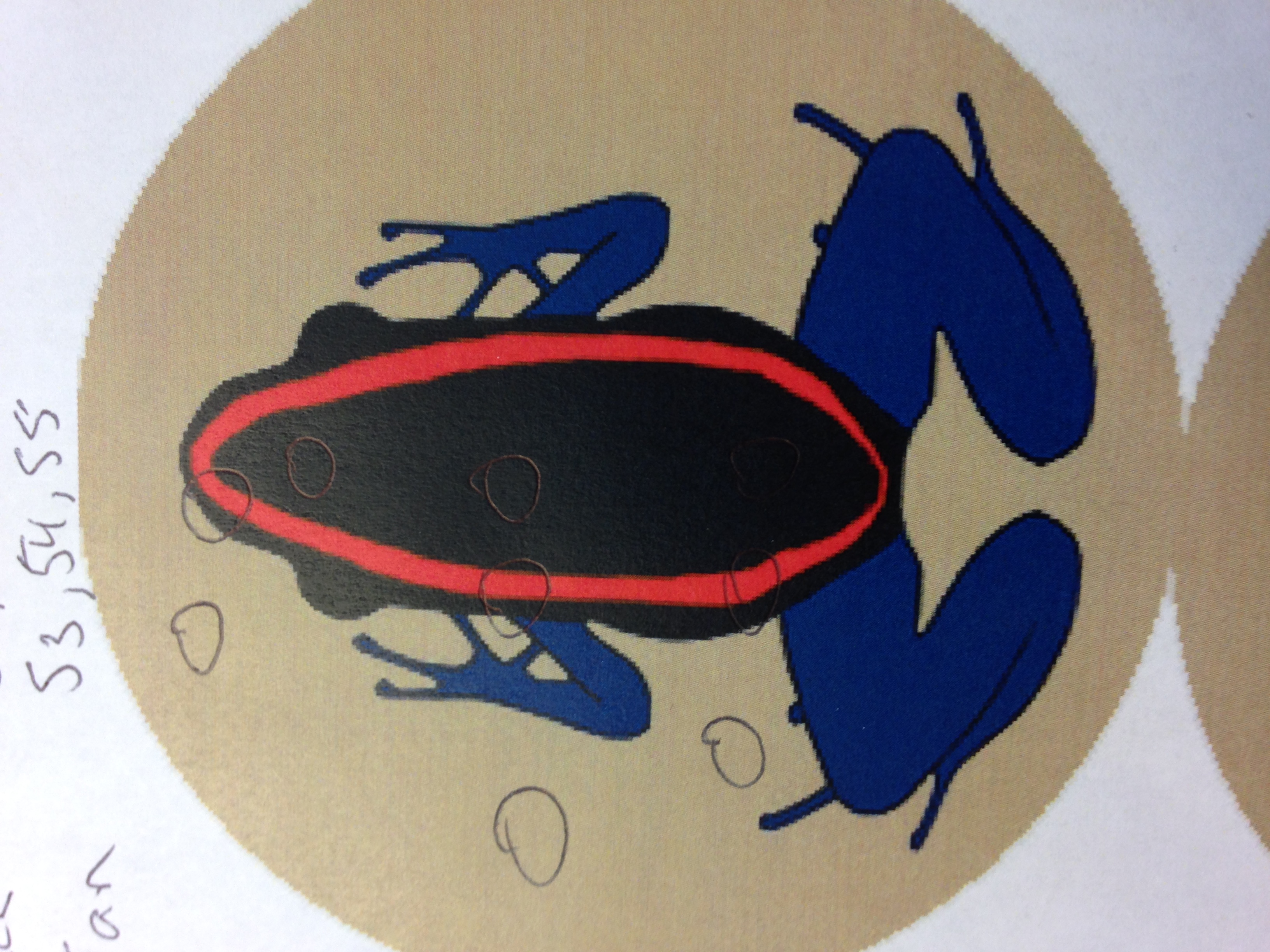

Supplement: S1 Text — (ZIP) [file pone.0194279.s001.zip › IMG_7336.JPG]

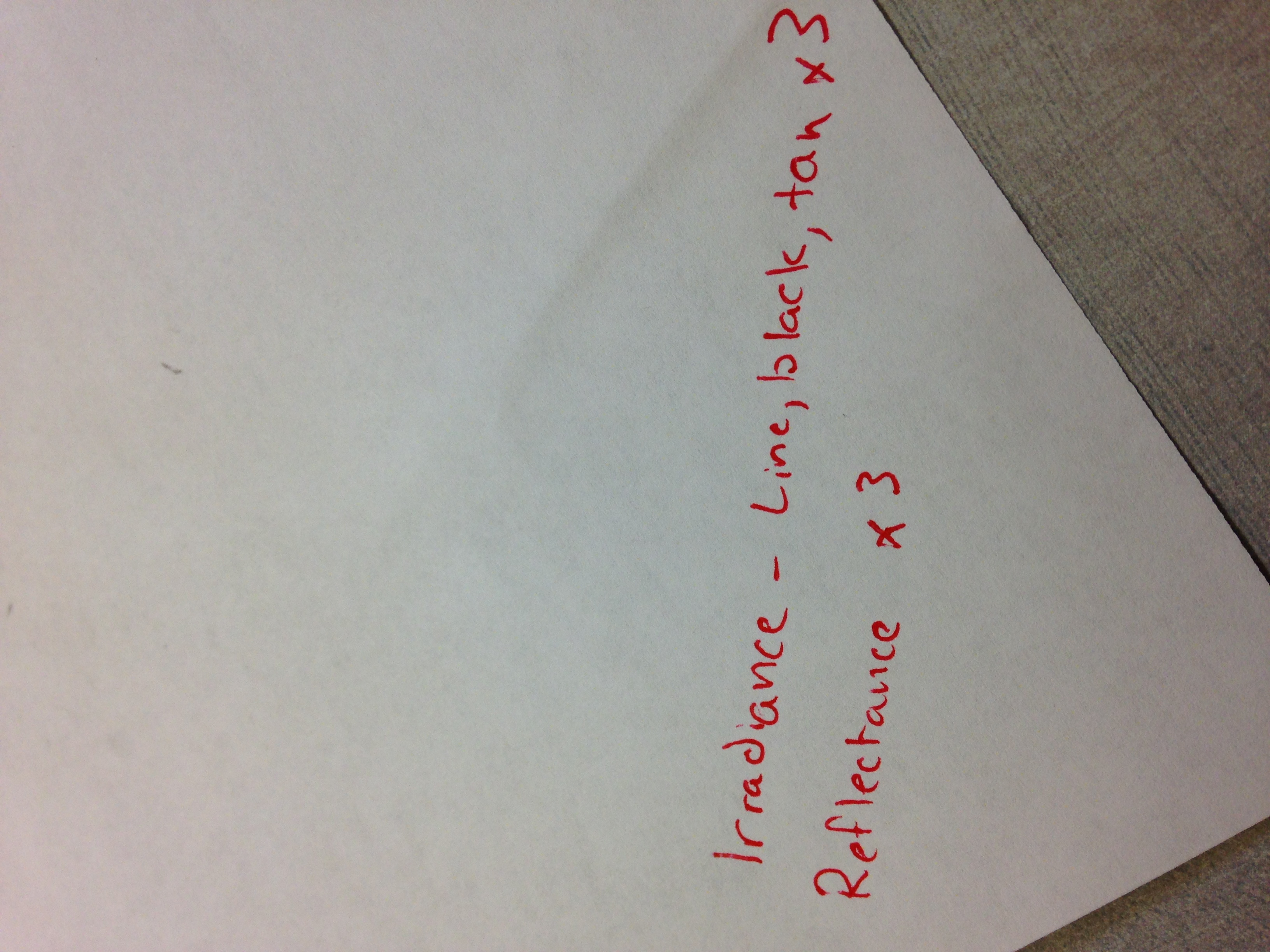

Supplement: S1 Text — (ZIP) [file pone.0194279.s001.zip › IMG_7337.JPG]
